# Interleukin-1beta-induced reduction of tissue water diffusion in the juvenile rat brain on ADC MRI is not associated with ^31^P MRS-detectable energy failure

**DOI:** 10.1186/s12950-016-0118-3

**Published:** 2016-03-17

**Authors:** Raman Saggu

**Affiliations:** MRC Biochemical and Clinical Magnetic Resonance Unit, Department of Biochemistry, South Parks Road, University of Oxford, OX1 3QU, Oxford, UK

**Keywords:** Interleukin-1beta, Magnetic resonance imaging, Apparent diffusion coefficient, Phosphorus magnetic resonance spectroscopy, Energy metabolism, Brain

## Abstract

**Background:**

It has long been known that an intrastriatal microinjection of the archetypal pro-inflammatory cytokine, interleukin-1beta (IL-1β), in juvenile rats induces a chronic reduction in the apparent diffusion coefficient (ADC) of tissue water on magnetic resonance imaging (MRI). Reduced ADC during acute cerebral ischaemia is an established indicator of metabolic failure whereas the cause of the IL-1β-induced reduction remains to be deciphered. Previously, it has been shown that IL-1β does not perturb the phosphorus (^31^P) magnetic resonance spectroscopy (MRS)-detectable energy status of an *ex vivo* preparation of rat brain parenchyma that is devoid of a functional vasculature component. However, brain energy status following an IL-1β challenge in vivo remains to be examined.

**Methods:**

This study is the first longitudinal in vivo examination of the correlation of ADC MRI with localised ^31^P MRS signals obtained specifically from within the injected and non-injected striatum following IL-1β (1 ng/ul or 100 ng/ul) challenge, in real-time.

**Results:**

Despite observing a chronic reduction in ADC at either dose of IL-1β challenge, energy compromise was not detected at any time point.

**Conclusions:**

The IL-1β-induced effects pertaining to a functional vasculature such as leukocyte recruitment, blood–brain barrier (BBB) breakdown and blood flow changes are unlikely to impact on overall tissue energy status. Compared to classic ischaemia, there is dissociation between ADC and energy status within an IL-1β-induced lesion in vivo.

## Background

Evidence suggests that the archetypal pro-inflammatory cytokine, interleukin-1beta (IL-1β), is a key component in the pathogenesis of stroke, Alzheimer’s disease (AD) [for review, see 1] and Parkinson’s Disease (PD) [for review, see 2]. These are complex pathologies in which it can be difficult to determine the physiological actions of IL-1β in the brain. However, by examining the consequences of a cerebral IL-1β challenge we may facilitate our understanding of the role of this cytokine in neurodegenerative disease and disorder.

Intrastriatal microinjection of IL-1β in three-week-old (P21) juvenile rats induces a chronic reduction in the apparent diffusion coefficient (ADC) of tissue water on magnetic resonance imaging (MRI) [3]. This is indicative of a loss of high-energy phosphates, detectable using non-invasive phosphorus (^31^P) magnetic resonance spectroscopy (MRS), in experimental acute cerebral ischaemia [4–6]. Mechanistically, a reduction in cerebral blood flow (CBF) promotes the failure of adenosine triphosphate (ATP)-dependent cell membrane ion pumps, dysregulating cell volume [for review, see [7], inducing cell swelling, reducing the extracellular space and, therefore, the ADC [8–10].

Contrary to classic ischaemia where ADC changes occur swiftly within a matter of minutes [11], IL-1β-induced changes do not present until 6 h post challenge [3]. Furthermore, IL-1β increases regional cerebral blood volume (rCBV), lactate levels remain unaltered [3], and there is an absence of neuronal cell death [12, 13]. It has been suggested that IL-1β-induced neutrophil-mediated blood-brain-barrier (BBB) breakdown may influence water diffusion within the rat brain parenchyma, affecting ADC changes [3]. However, BBB breakdown occurs between 4 h and 5 h and gadolinium post-contrast enhancement diminishes by 6 h when ADC first becomes significantly reduced [3]. Furthermore, the BBB reseals itself by 24 h whereas ADC changes persist up to 123 h, and are not prevented by neutrophil depletion [3]. IL-1β-induced morphological changes such as microglial activation or cell swelling have also been suggested as possible explanations for the reduced ADC [12]. Electrical impedence measurements in a rat model of NMDA-induced excitotoxic injury have shown that an ~10 % cell swelling corresponds to an ~50 % reduction in ADC [10]. However, microglia revert to resting morphology 72 h post IL-1β challenge whilst ADC remains depressed beyond this time point [3]. Thus, although ischaemia, BBB changes and cell swelling do not seem to account for the ADC reduction observed in this model, IL-1β-induced energy failure in vivo remains a possibility.

Reduced ADC is observed in rat models of *N*-methyl-D-aspartate (NMDA)- [14] or ouabain-induced [15] excitotoxic injury, and bicuculline-induced status epilepticus [16], which exhibit no apparent ischaemic component to their pathology. Nevertheless, these studies sought to correlate ADC changes with the energy status of the brain given the strong association between failure of energy-dependent transmembrane Na^+^/K^+^-ATPase ion pumps and dysregulation of cell volume [for review, see 7]. Therefore, the metabolic status of brain parenchyma following IL-1β challenge in vivo warrants examination to determine whether an ischaemia-independent energy deficit may be associated with the reduction in ADC.

IL-1β does not appear to affect high-energy phosphate metabolism of an *ex vivo* preparation of brain parenchyma *per se* [17]. ^31^P MRS was used to characterise the energy status of organotypic hippocampal-slice cultures (OHSCs) prior to and following IL-1β challenge. The OHSC set-up preserves brain parenchyma cytoarchitecture and synaptic connectivity without the complication of a functional vascular component. Thus, the vascular-related consequences of an IL-1β challenge in vivo such as neutrophil recruitment, blood–brain barrier (BBB) and perfusion changes [3] can be extricated from those pertaining to the parenchyma.

The question of whether IL-1β affects cerebral energy metabolism remains partly addressed; tissue energy status should additionally be addressed in vivo where the physiological consequences of a functional vasculature are included. Furthermore, the classic bio-imaging marker of an energy deficit - a reduced ADC - may only be observed, followed and correlated with metabolism in real-time, in vivo.

Herein, the energetic contribution to the IL-1β-induced reduction in ADC in vivo was examined. A comprehensive characterisation of the real-time longitudinal evolution of the lesion on ADC MRI is presented. Localised ^31^P MRS-detectable energy status, as determined by the phosphocreatine (PCr) to ATP ratio [18], was measured within the ipsilateral and contralateral striatum 6 h following intrastriatal microinjection of 1 ng/μl IL-1β when ADC first becomes reduced at this dose [3]. Additionally, ^31^P MRS, ADC and anatomical [T_1_-weighted (T_1_-w)/T_2_-weighted (T_2_-w)] MRI data were acquired serially in the same animal at 2.5 h, 6 h, 24 h and 72 h following an intrastriatal 100 ng/μl IL-1β challenge.

## Methods

### Reagents

Rat recombinant (rr) cytokine, interleukin-1beta (rrIL-1β) (NIBSC, Potters Bar, UK) was dissolved in endotoxin-free saline (vehicle).

### Animal preparation

Three-week-old male juvenile Wistar rats (Harlan-Olac) were used to examine the effects of an intrastriatal IL-1β challenge, as reported previously [3]. Animals were anaesthetised by inhalation using 2.0 % isoflurane in 70 % N_2_O: 30 % O_2_ and positioned within a stereotaxic frame. Under microscope guidance, a burr hole was made in the skull 3 mm caudal and 1 mm lateral to Bregma in the left hemisphere. Using a finely drawn glass micropipette, 1 μl injection of the IL-1β solution (1 ng/μl or 100 ng/μl) was delivered at a depth of 4 mm from the brain surface over a 5 min period. Control animals were injected with saline. Animals were placed in a purpose-built head restraint system comprised of a tooth bar and a nose strap integral to the coil. A 2 cm-diameter, single-turn, ^31^P-tuned (32.5 MHz) circular surface coil, bent to conform to the curve of the skull, was positioned over the animal’s head and used for both excitation and signal acquisition. Anaesthesia was maintained within the magnet using 1.0 % isoflurane in 70 % N_2_O: 30 % O_2_ administered via tubing positioned around the animal’s snout. All procedures were carried out with ethical approval under a UK Home Office Licence.

### Magnetic resonance methods

MR measurements were made using a 300 MHz (7 Tesla) horizontal bore magnet (Oxford Instruments, Oxford, UK), internal bore size 10.2 cm. Data acquisition, gradient and waveform generation, and ancillary processes were carried out on a Varian Inova System spectrometer (Varian Medical Systems, Palo Alto, CA, USA).

#### Proton MR Probe

Radiofrequency (RF) pulses were applied using a 3.4 cm Alderman-Grant resonator (4.2 cm in length) secured inside an custom-built cylindrical Perspex probe body (34 cm in length; aluminium base plate: 21 cm in length). The internal dimensions of the coil closely matched the size of the animal’s head, thereby minimising noise acquisition from areas other than the region of interest. An earthed copper shield was positioned around the RF coil to prevent coupling between the probe and gradients. The RF coil was part of a tunable resonance circuit.

#### Anatomical imaging

Anatomical (T_1_-weighted and T_2_-weighted/T_1_-w and T_2_-w) images were acquired using a fast spin-echo sequence. T_1_-w images used a repetition time (TR) of 0.5 s, and an echo time (TE) of 0.02 s whereas T_2_-w images used a TR of 3.0 s and a TE of 0.04 s. Slice thickness, 1 mm; field of view (FOV), 3 × 3 cm^2^; matrix size, 128 × 128 pixels with nominal in-plane resolution, 0.23 × 0.23 mm^2^.

#### Diffusion-weighted imaging

Diffusion-weighted images (DWI) were acquired using a navigated pulsed-gradient spin-echo sequence with a TR of 2 s and a TE of 0.0365 s. Imaging was performed using the three diffusion-weighting (“b”) values of 125, 500 and 1000 s/mm^2^, where *b* is defined by the equation *b* = (*γG*δ)^2^[*Δ* − δ/3] [19]. *G* is the diffusion gradient strength; *Δ*, the diffusion time, was 17.5 ms and δ, the diffusion gradient duration, was 12.5 ms. Diffusion gradients were applied simultaneously on all three axes, and “trace” diffusion measurements were calculated [20]. Motion correction was addressed using navigator echoes [21]. Slice thickness, 1 mm; FOV, 3.5 × 3.5 cm^2^ and matrix size, 128 × 128 pixels.

#### Phosphorus magnetic resonance spectroscopy

Localised phosphorus MR spectra were acquired from a cuboidal volume (5 × 6 × 4 mm^3^) positioned within the striatum of each hemisphere. An image-selected in vivo spectroscopy (ISIS) sequence was used to minimise signal contamination from other brain regions (TR, 6 s; transient number, 600; TE, 0.01 s; number of points, 6144; total acquisition time, 1 h 56 s; bandwidth, 6000 Hz). Proton decoupling was applied.

### Experimental protocol

Each animal was injected with IL-1β or vehicle at time 0 and serially imaged 2.5 h, 6 h, 24 h and 72 h post challenge. Animals were allowed to recover after the 6 h, 24 h and 72 h time points. T_2_-w, T_1_-w, ADC MRI and ^31^P MRS data were acquired for each animal and the time point for each image was taken to be the mid-point of the image acquisition time. ^31^P MRS data for the contralateral hemisphere at 6 h was not obtained as this would have prolonged the extensive (10 h) protocol on day 1 of imaging by a further 1.5 h. Maintaining the animal under anaesthesia for long periods risks death and changes in energy metabolism were not expected to be observed in the contralateral hemisphere at this time point.

MRI: vehicle-injected animals, *n* = 3 at each time point; 100 ng/μl IL-1β-injected animals, *n* = 6 at each time point. ^31^P MRS: vehicle-injected animals, *n* = 3 at each time point; 100 ng/μl IL-1β-injected animals, *n* = 6 at 2.5 h, *n* = 5 at 6 h, *n* = 6 at 24 h, *n* = 4 at 72 h ipsilateral hemisphere and *n* = 5 at 2.5 h, *n* = 4 at 24 h, *n* = 5 at 72 h contralateral hemisphere; 1 ng/μl IL-1β-injected animals, *n* = 4 at each time point.

### Magnetic resonance data analysis

#### ADC MRI

##### Quantitation of ADC perturbation

ADC images were generated from diffusion-weighted images [22]. Reduction in ADC was quantified by: 1) Drawing a region of interest (ROI) onto ADC maps, using a freehand cursor, that encompassed the striatum of the contralateral hemisphere of each animal at each time point, and attaining an average value for the SD of the ADC. 2) Thresholding each ADC map by subtracting 1 times the average SD from the actual ADC value of each animal at each time point. Previously, Blamire and colleagues reported thresholding ADC maps to 1 SD below the mean for analysis following intrastriatal microinjection of 1 ng/μl IL-1β [3]. 3) Drawing an ROI around the areas highlighted by thresholding within the injected hemisphere. 4) Mapping the ROI back onto original ADC maps to attain the ADC value, which also gives the ROI area. 5) Flipping the ROI of the injected hemisphere on the original ADC map onto the contralateral hemisphere to attain an ADC value for the non-injected hemisphere.

##### Measuring ADC within an ROI encompassing the dimensions of the ^31^P MRS voxel

In addition to drawing an ROI around the area highlighted by thresholding and mapping back onto original ADC maps, separate ADC values were obtained for the ROI encompassing the dimensions of the ^31^P MRS voxel within the striatum of the ipsilateral and contralateral hemispheres.

##### ^31^P MRS

Spectra were fitted in the time domain using a nonlinear least squares algorithm (AMARES) [23, 24]. The phosphocreatine (PCr) to gamma (γ) adenosine triphosphate (ATP) ratio was used as a measure of tissue energy status [18].

### Statistics

Statistical analysis was performed using student *t*-test or repeated measures analysis of variance (ANOVA) test.

## Results

### MRI

#### Thresholding demonstrates a significantly reduced ADC that presents at 6 h and persists up to 72 h

**Fig. 1:**
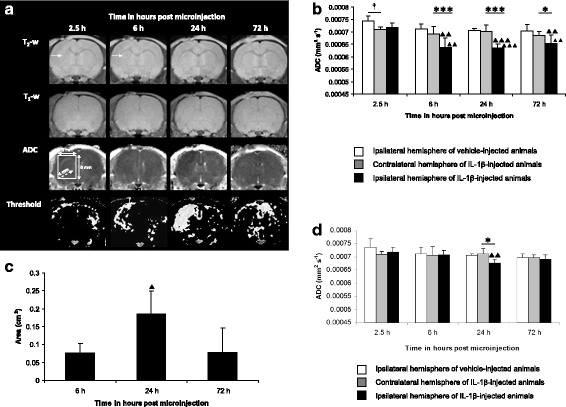
Time course of MRI changes following intrastriatal microinjection of 100 ng/μl IL-1β. **a** *Representative T*_*2*_ *-w and T*_*1*_ *-w images, ADC maps and their respective thresholded images from the same animal (1 SD below mean ADC of contralateral hemisphere of cohort imaged at 2.5 h, 6 h, 24 h and 72 h).* Arrow points to injection site, visible as a focal area of T_2_-w hyperintensity at 2.5 h and 6 h, and reflects the volume of fluid injected. **b** *ADC changes determined by thresholding*. Mean ADC (error bars indicate a 1 SD) within the ipsilateral or contralateral striatum. Statistical significance indicated by *** *p* < 0.001, * *p* < 0.05; paired *t*-test, † *p* < 0.05; unpaired *t*-test and ▲▲▲ *p* < 0.001, ▲▲ *p* < 0.01; one-way ANOVA, post-testing using Bonferroni multiple comparisons test, with respect to the ADC of the IL-1β-injected hemisphere at 2.5 h **c** *Evolution of area of reduced ADC.* Mean area (error bars indicate a 1 SD) within the injected hemisphere. Statistical significance indicated by ▲ *p* < 0.05; one-way ANOVA, post-testing using Bonferroni multiple comparisons test, with respect to the area of reduced ADC at 6 h. **d** *ADC measured within the dimensions of the* ^*31*^ *P MRS voxel* (position and dimensions shown in Fig. 1a). Mean ADC (error bars indicate a 1 SD) within a region of interest encompassing the ^31^P MRS voxel within the ipsilateral or contralateral striatum. Statistical significance indicated by * *p* < 0.05; paired *t*-test ▲▲ *p* < 0.01; one-way ANOVA, post-testing using Bonferroni multiple comparisons test, with respect to the ADC of the IL-1β- injected hemisphere at 2.5 h. Vehicle- (saline) injected animals (*n* = 3 at each time point) and IL-1β-injected animals (*n* = 6 at each time point)

One-way ANOVA showed significant variation in ADC of the IL-1β-injected hemisphere (*p* = 0.0004), and post-testing using Bonferroni multiple comparisons test showed a significantly reduced ADC at 6 h (*p* < 0.01), 24 h (*p* < 0.001) and 72 h (*p* < 0.01) compared with 2.5 h (Fig. 1a and b).

#### The area of reduced ADC, identified by thresholding, is spatially greatest at 24 h

One-way ANOVA showed significant variation in the area of reduced ADC from 6 h to 72 h (*p* = 0.0063), and post-testing using Bonferroni multiple comparisons test showed a significantly greater area at 24 h compared with 6 h (*p* < 0.05) (Fig. 1c).

#### With respect to the dimensions of the ^31^P MRS voxel, the ADC change is significant at 24 h only, and returns to baseline by 72 h

One-way ANOVA showed significant variation (*p* = 0.0034) in the ADC of tissue contained within the ^31^P MRS voxel. Post-testing using Bonferroni multiple comparisons test showed significantly reduced ADC at 24 h compared with 2.5 h (*p* < 0.01) (Fig. 1d). By 72 h, there was no significant difference in ADC between the IL-1β- and vehicle-injected hemispheres (*p* = 0.6151; unpaired *t*-test).

#### The ADC of the ROI at 6 h (ROI_6 h_) remains persistently depressed throughout the course of the study

**Fig. 2:**
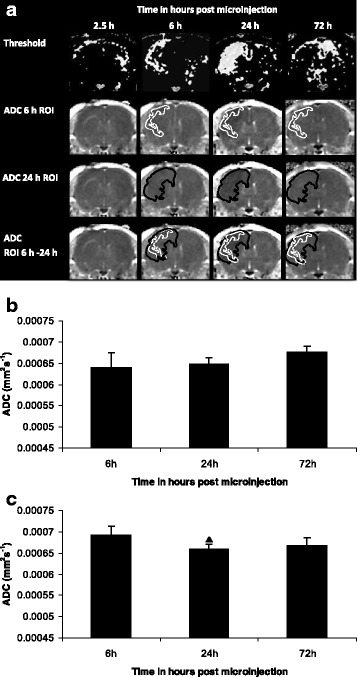
ADC ROI mapping. **a** The ROI of reduced ADC, identified by thresholding, when it first becomes reduced (ROI_6 h_) was mapped forwards onto original ADC maps at subsequent time points (in white). The ROI of reduced ADC at 24 h (ROI_24 h_), the greatest area of ADC change, was mapped onto 6 h and 72 h ADC maps (in black). The ROIs of 6 h and 24 h time points are overlaid in the last row; the spatial difference representing the ADC of ROI_6 h-24 h_. All images are from the same animal and are representative of the group. **b** Time course of development of the ADC of the ROI_6 h_. Mean ADC (error bars indicate a 1 SD). **c** Time course of development of the ROI_6 h-24 h_ *.* Mean ADC (error bars indicate a 1 SD). Statistical significance indicated by ▲ *p* < 0.05; one-way ANOVA, post-testing using Bonferroni multiple comparisons test, with respect to the ADC at 6 h. IL-1β-injected animals, *n* = 6 at each time point

One-way ANOVA showed significant variation in the ADC of ROI_6 h_, *p* = 0.0416, although post-testing by Bonferroni multiple comparisons test did not, suggesting that the area of reduced ADC at 6 h remains depressed throughout the course of the study (Fig. 2a and b).

#### The ADC of the spatial difference between the initial ROI (ROI_6 h_) and the ROI of greatest spatial extent at 24 h (ROI_24 h_) decreases further between 6 h and 24 h

The ADC of the spatial difference between the 6 h and 24 h ROI (ROI_6 h-24 h_) became further reduced at 24 h compared with 6 h as demonstrated by one-way ANOVA, *p* = 0.0154 and post-testing using Bonferroni multiple comparisons test (Figs. 2a and c).

### ^31^P MRS

**Fig. 3:**
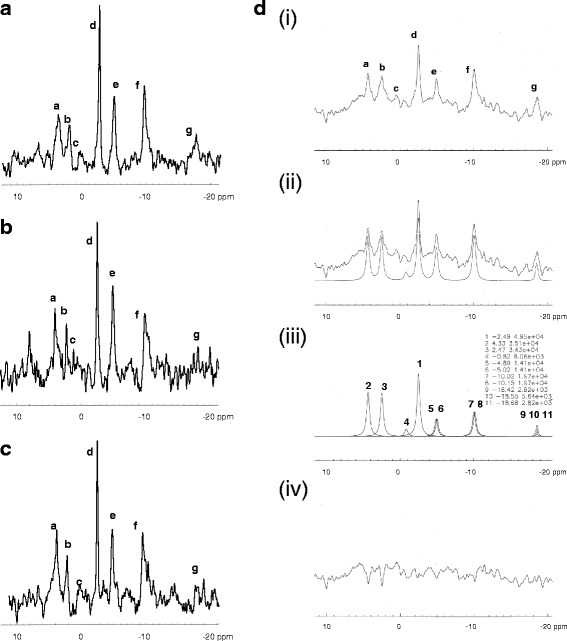
Representative in vivo ^31^P MR spectra from **a** the ipsilateral striatum of a 1 ng/μl IL-1β-injected animal, **b** the ipsilateral striatum of a 100 ng/μl IL-1β-injected animal and **c** the contralateral striatum of a 100 ng/μl IL-1β-injected animal. Each spectrum exhibits peaks from the phosphomonoesters (PME, a); inorganic phosphate (Pi, b); the phosphodiesters (PDE, c); phosphocreatine (PCr, d) and the three resonances of the gamma (γ, e), alpha (α, f) and beta (β, g) phosphates of adenosine triphosphate (ATP). **d** Representative ^31^P MR spectrum of the ipsilateral striatum of a saline-injected animal showing integration of the spectral peaks. (i) Original spectrum. (ii) Baseline correction and spectral fit using a software package compiling the AMARES algorithm [23, 24]. (iii) γATP and αATP are doublets and comprised of two resonances of equal area, γ: peaks 5 and 6, α: peaks 7 and 8 respectively. βATP is a triplet comprised of three resonances; two of which are of equal area: peaks 9,10 and 11. PCr (peak 1) is a singlet. (iv) The spectral residue (spectrum minus fit).

^31^P MR spectra exhibited the prime high-energy phosphates: the phosphomonoesters, PME; inorganic phosphate, Pi; phosphocreatine, PCr and adenosine triphosphate, ATP. ATP comprises three resonances, one arising from each of the phosphorus atoms in the ATP molecule: gamma (γ), alpha (α) and beta (β)ATP (Fig. 3). Each resonance is a multiplet determined by the number of neighbouring phosphorus nuclei influencing the magnetic field of each ^31^P nucleus: βATP being a triplet and the γATP and αATP resonances doublets. Spectra were centred (transmission off-resonance, tof) halfway between the PCr and γATP resonances to enable optimal resolution of these peaks, which are required for calculating the energy status (PCr to γATP ratio); hence the relatively poor phasing of the PME, Pi and βATP peaks.

#### Intrastriatal microinjection of 1 ng/μl IL-1β induces a reduction in ADC at 6 h that is not accompanied by a ^31^P MRS-detectable reduction in the PCr to γATP ratio

**Fig. 4:**
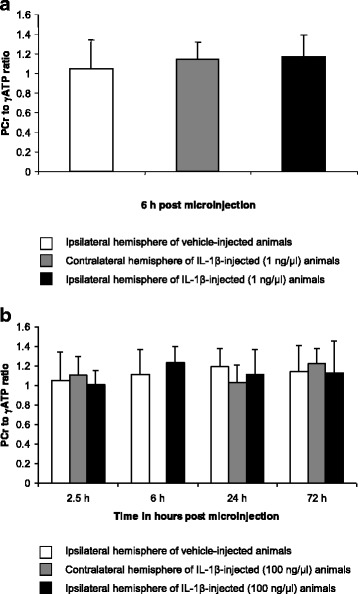
The ^31^P MRS-detectable energy status of brain parenchyma following IL-1β challenge*.* **a** Histogram showing the mean PCr to γATP ratio (error bars indicate a 1 SD) within the ipsilateral and contralateral striatum of IL-1β-injected (1 ng/μl) animals (*n* = 4) and the ipsilateral striatum of vehicle- (saline) injected animals (*n* = 3) at 6 h. **b** Histogram showing the mean PCr to γATP ratio (error bars indicate a 1 SD) within the ipsilateral and contralateral striatum of IL-1β-injected (100 ng/μl) animals and the ipsilateral striatum of vehicle- (saline) injected animals. ^31^P MRS data was not obtained for the contralateral hemisphere at 6 h as this would have prolonged the extensive (10 h) protocol on day 1 of imaging by a further 1.5 h. Maintaining the animal under anaesthesia for long periods risks death and changes in energy metabolism were not expected to be observed in the contralateral hemisphere at this time point. IL-1β-injected animals: *n* = 6 at 2.5 h, *n* = 5 at 6 h, *n* = 6 at 24 h, *n* = 4 at 72 h for the ipsilateral hemisphere and *n* = 5 at 2.5 h, *n* = 4 at 24 h and *n* = 5 at 72 h for the contralateral hemisphere. Vehicle- (saline) injected animals: *n* = 3 at each time point

There was no difference in the mean PCr to γATP ratio (Fig. 4a) of the IL-1β-injected ipsilateral and contralateral hemispheres at 6 h (*p* = 0.7890, *n* = 4, paired *t*-test), or in the mean PCr to γATP ratio of the IL-1β- and vehicle-injected ipsilateral hemispheres at 6 h (*p* = 0.3243, *n* = 3, unpaired *t*-test).

#### Intrastriatal microinjection of 100 ng/μl IL-1β induces a chronic reduction in ADC that is not associated with a perturbation in the PCr to γATP ratio at any time point (2.5 h, 6 h, 24 h or 72 h)

There was no significant variation in the PCr to γATP ratio of the IL-1β-injected ipsilateral (one-way ANOVA, *p* = 0.4824) or contralateral hemisphere (one-way ANOVA, *p* = 0.2526). Paired *t*-test showed no significant difference in the PCr to γATP ratio of the IL-1β-injected ipsilateral and contralateral hemispheres at 2.5 h (*p* = 0.3434), 24 h (*p* = 0.6347) or 72 h (*p* = 0.6434) (Fig. 4b).

### Power calculations

Power analysis for the PCr to γATP ratio of the IL-1β-injected ipsilateral versus contralateral hemisphere, paired *t*-test at 24 h was performed according to Rosner (2011) [25]. This gave a sample estimate of *n* = 49 in each group (injected and contralateral hemisphere) needed to observe a change in the PCr to γATP ratio of at least 25 %; type I error (α) = 0.05, type II error (β) = 20 %. The equations applied were:

1 $$ {\upsigma_{\mathrm{d}}}^2\kern0.5em =\kern0.5em {\upsigma_2}^2+{\upsigma_1}^2{\textstyle \hbox{-} }2{\uprho \upsigma}_1{\upsigma}_2\kern0.5em \mathrm{where}: $$

ρ = Pearson correlation coefficient;

σ_1_ = standard deviation for the contralateral hemisphere at 24 h;

σ_2_ = standard deviation for the injected hemisphere at 24 h;

2 $$ \mathrm{n}\kern0.5em =\kern0.5em \frac{{{2\upsigma}_{\mathrm{d}}}^2{\left({\mathrm{z}}_{1\hbox{-} \upalpha /2}+\kern0.5em {\mathrm{z}}_{1\hbox{-} \upbeta}\right)}^2}{{\left[{\upmu}_1-{\upmu}_2\right]}^2}\kern0.5em \mathrm{where}: $$

μ_1_ = mean value of the contralateral hemisphere at 24 h;

μ_2_ = mean value of the injected hemisphere at 24 h;

z_q_ = q^th^ percentile of a normal distribution.

### Vehicle imaging changes

T_1_-w imaging did not detect any effects of IL-1β or vehicle microinjection. A focal area of hyperintensity was visible on T_2_-w imaging 2.5 h following either IL-1β or vehicle microinjection, which resolved thereafter, and is likely due to the volume of fluid injected. Vehicle-injected animals exhibited elevated ipsilateral ADC compared with the contralateral hemisphere at 2.5 h, due to the volume of saline injected, which transiently raises the ADC. ^31^P MRS of vehicle-injected animals demonstrated no change in energy status at any time point.

## Discussion

In the present study, the energetic contribution to the IL-1β-induced reduction in ADC was examined in vivo, for the first time. A potential energy deficit was hypothesised since failure of the ATP-dependent transmembrane ion pumps [for review, see 7] is critical in controlling the volume of the intra- and extracellular space, and thus the ADC [8–10].

An IL-1β-induced (100 ng/μl) reduction in ADC was observed from 6 h to 72 h post challenge (as determined by thresholding), and was due to a combination of the ADC of the initial ROI at 6 h (ROI_6 h_) remaining depressed throughout the study (Fig. 2b) and the ADC of the spatial difference between ROI 6 h and ROI 24 h (ROI_6 h-24 h_) becoming further depressed (Fig. 2c). However, the ADC within the dimensions of the ^31^P MRS voxel was reduced only at 24 h (Fig. 1d), coinciding with the greatest spatial extent of ADC change (Fig. 1c), and returned to baseline by 72 h (Fig. 1d). The difference in sensitivity with respect to both analyses is important, as the ADC changes detected by thresholding are spatially smaller than the dimensions of the ^31^P MRS voxel (Fig. 1a). If an IL-1β-induced reduction in ADC is indeed associated with energy failure, the spatial extent of ADC changes at 6 h and 72 h may not be sufficient to correlate with ^31^P MRS-detectable perturbations in the PCr to γATP ratio. However, the large spatial extent of ADC change at 24 h (Fig. 1a and c) in parallel with a significantly reduced ADC (Fig. 1b and d) suggests that one should, at least, be able to detect energy failure at this time point.

Interestingly, ^31^P MRS did not detect a change in the PCr to γATP ratio at any time point following 100 ng/μl IL-1β challenge (Fig. 4b), nor at 6 h following 1 ng/μl IL-1β challenge (Fig. 4a) when ADC is first reported to become reduced at this dose [3]. This finding is in agreement with previous observations using an *ex vivo* preparation of rat brain parenchyma that demonstrated an unperturbed ^31^P MRS-detectable energy status 6 h following incubation with IL-1β [17].

In vivo localised rat brain ^31^P MRS is a technically challenging method and was used in the present study to measure the tissue energy status specifically within the area of IL-1β microinjection, minimising contamination of signal from surrounding non-injected tissue. However, this approach has sensitivity limitations. The dimensions of the ^31^P MRS voxel must be sufficiently large for signal detection within a reasonable time acquisition (in this case, 1 h). In the present study, the mean peak height for PCr within the left and right cerebral hemispheres for 3 control animals was 85 mm, and the standard deviation 19 mm. Thus, within the limits of sensitivity of this protocol, a minimal reduction of approximately 25 % in PCr is required to detect a change in the PCr to γATP ratio.

One should consider how large a reduction in the PCr to γATP ratio might be required to constitute energy failure with confidence. In cerebral ischaemia in the rat, in vivo PCr and ATP levels measured by ^31^P MRS have been shown to decrease in parallel [26], in which case one would not be able to detect an overall change in the the PCr to γATP ratio. However, these observations were based on extreme ends of the spectrum where the metabolites were either nearing control values or were extremely compromised [27–29]. Moderate ischaemic challenges where ^31^P MRS-detectable PCr is reduced but ATP is maintained have demonstrated that PCr must decrease to 40 ± 26 % before ATP loss occurs [30]. When PCr is above 25 % of control value, ATP loss is one-third that of PCr whereas below 25 % of control PCr value, ATP loss supersedes that of PCr [30].

A better estimate of the sensitivity of the ^31^P MRS protocol may be provided by a power calculation. The sample size required to detect a 25 % change in the PCr to γATP ratio 24 h following 100 ng/μl IL-1β challenge, the time point exhibiting the spatially greatest and severest reduction in ADC, is *n* = 49. This is impractical given the challenging nature and long duration of the ^31^P MRS protocol, and reinforces the unlikelihood that IL-1β induces energy failure in vivo. By comparison, in rat cerebral ischaemia, whilst PCr is above 25 % of control value, ATP loss is one-third that of PCr [30], as explained earlier. If, on that basis, a power calculation is performed to show how many animals are required to demonstrate a 75 % change in the PCr to γATP ratio, *n* = 5. Therefore, within the sensitivity limitations of the ^31^P MRS protocol, it is likely that a change in the PCr to γATP ratio would only be detectable if the metabolic impact was severe enough, which does not appear to be the case following IL-1β challenge.

It is important to address the correlation between ADC reduction and energy failure using in vivo models. In the classic middle cerebral artery occlusion (MCAO) model of brain ischaemia, energy failure perturbs ionic gradients inducing cell oedema and reducing the extracellular space, which depresses the ADC to 60–70 % of the original value by 7 h [6, 31–33]. The association between ADC changes and metabolic failure following MCAO has been proven by anatomic mapping of regions of depressed ADC on MRI with areas of acidosis, lactate accumulation [34] and ATP loss, assessed using bioluminescence imaging on frozen tissue [6]. One may conclude that a decrease in ADC of approximately 40 % of its control value or the value in the non-injected contralateral hemisphere constitutes energy failure in MCAO.

The same reasoning cannot be translated to other non-ischaemic examples of ADC reduction in vivo. For example, an excitotoxic NMDA challenge in the rat brain induces a comparable ~ 40 % reduction in ADC, which affects almost the entire injected hemisphere, without tissue energy failure as assessed by ^31^P MRS [14]. Injection of the excitotoxin, ouabain, into the rat brain produces an approximate 40 % drop in ADC, albeit within a smaller sized lesion but without ^31^P MRS-detectable metabolic failure [15]. Interestingly, brain ADC reductions of ~ 18 % have been reported in an experimental model of status epilepticus, which did not correlate with energy failure [16]. Thus, given our present incomplete understanding of the mechanisms of in vivo ADC reduction, it is not possible to make a direct association between magnitude of ADC reduction and prediction of tissue energy failure in models other than MCAO.

Partial volume effects are significant with respect to both ADC and ^31^P MRS measurements. Following IL-1β challenge, the partial volume effects on ADC measurement have been demonstrated by comparing the ROI analysis with the ^31^P MRS voxel analysis (Fig. 1b and d, respectively). In Fig. 1b, the ADC at 24 h is reduced within the injected hemisphere to ~ 90 % of the value in the contralateral hemisphere. In Fig. 1d, the ADC measurement within the confines of the ^31^P MRS voxel detects an ADC reduction to 95 % of the value in the contralateral hemisphere, thus halving the magnitude. IL-1β diffusibility may appear unrestricted since ADC changes were observed throughout the cerebral hemisphere, particularly at 24 h, however, it seems that only 50 % of the parenchyma contained within the ^31^P MRS voxel was affected by tissue water diffusion changes, which would be expected to affect the sensitivity of ^31^P MRS-detectable changes. However, as discussed earlier, one cannot draw a direct correlation between magnitude of ADC reduction and energy failure in non-ischaemic challenges.

It is of note that astrocytes are the only brain cell-type to exhibit a glycogen reserve, utilised during ischaemia to preserve neuronal viability [35]. Interestingly, challenging dissociated primary neuronal or astrocytic cell cultures with IL-1β induces astrocytic utilisation of their endogenous glycogen reserve whilst neuronal metabolism is unaltered [36, 37]. Hence, an IL-1β challenge in vivo may not alter the overall PCr to γATP ratio of brain parenchyma.

The present study has shown that the ADC reduction cannot be confidently attributed to depleted ATP levels, as measured by ^31^P MRS. This finding has important implications for the interpretation of ADC changes in the brain because it occurs under metabolic conditions distinct from those concerning classic cerebral ischaemia, as is the case with rodent models of excitotoxic injury [14, 15] and status epilepticus [16]. There are clear differences in the consequences of an ischaemic, excitotoxic or IL-1β-induced challenge for neurons. Ischaemic or excitotoxic challenge induces neuronal cell death [14, 15] whereas an intrastriatal IL-1β microinjection is not neurotoxic [12, 13]. Therefore, any mechanistic explanation for reduced ADC following IL-1β challenge is likely to be different compared to that following excitotoxic injury or ischaemia.

An improved understanding of the mechanisms underlying brain ADC changes in non-ischaemic challenge and a closer examination of non-ischaemic in vivo models of ADC reduction are warranted as this may facilitate an improved understanding of brain pathophysiology observed non-invasively by routine ADC MRI. This is particularly pertinent at the present time as IL-1β appears to be a key player in the pathogenesis of neurodegenerative diseases such as Alzheimer’s Disease and Parkinson’s Disease [for reviews, see 1, 2]. There is great interest in MR examination of these diseases clinically and pre-clinically, particularly with respect to tissue water diffusion measurements [38–40]. In order to interpret the MR results effectively, it would be useful to have an improved understanding of the contribution of the IL-1β-induced component of the pathology to the brain tissue water diffusion signal.

## Conclusions

This is the first reported ^31^P MRS investigation of the energetic response of brain parenchyma to an intrastriatal IL-1β challenge in vivo. Energy status, as defined by the PCr to γATP ratio, remained unaltered within the limits of localised ^31^P MRS detection, despite a chronically reduced ADC classically associated with energy failure in acute cerebral ischaemia. The negative finding of no change in this energetic parameter is significant because it has been an unaddressed question in the field for over a decade. Previous works investigating BBB breakdown [3] and microglial activation [12] have also failed to account for the IL-1β-induced chronic reduction in ADC. However, it is only by investigation of these parameters that one may exclude them as causal factors.

## Abbreviations

IL-1β: Interleukin-1beta
MRI: Magnetic resonance imaging
ADC: Apparent diffusion coefficient
^31^P MRS: Phosphorus magnetic resonance spectroscopy
BBB: Blood–brain barrier
ATP: Adenosine triphosphate
PCr: Phosphocreatine
γ: Gamma
CBV: Cerebral blood volume
