# Erratum to: Interleukin-1beta-induced reduction of tissue water diffusion in the juvenile rat brain on ADC MRI is not associated with ^31^P MRS-detectable energy failure

**DOI:** 10.1186/s12950-016-0124-5

**Published:** 2016-05-23

**Authors:** Raman Saggu

**Affiliations:** MRC Biochemical and Clinical Magnetic Resonance Unit, Department of Biochemistry, University of Oxford, South Parks Road, Oxford, OX1 3QU UK

Unfortunately, after publication of this article [1], it was noticed that Fig. 1b (Fig. 1 here) is incorrect. The corrected figure can be seen below.

**Fig. 1:**
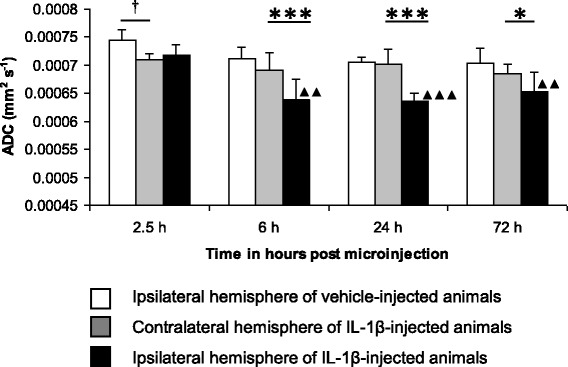
Time course of MRI changes following intrastriatal microinjection of 100 ng/μl IL-1β. *ADC changes determined by thresholding*. Mean ADC (error bars indicate a 1 SD) within the ipsilateral or contralateral striatum. Statistical significance indicated by *** *p* < 0.001, * *p* < 0.05; paired *t*-test, † *p* < 0.05; unpaired *t*-test and ▲▲▲ *p* < 0.001, ▲▲ *p* < 0.01; one-way ANOVA, post-testing using Bonferroni multiple comparisons test, with respect to the ADC of the IL-1β-injected hemisphere at 2.5 h
